# Markov Transition Field Combined with Convolutional Neural Network Improved the Predictive Performance of Near-Infrared Spectroscopy Models for Determination of Aflatoxin B_1_ in Maize

**DOI:** 10.3390/foods11152210

**Published:** 2022-07-25

**Authors:** Bo Wang, Jihong Deng, Hui Jiang

**Affiliations:** School of Electrical and Information Engineering, Jiangsu University, Zhenjiang 212013, China; wangbo@ujs.edu.cn (B.W.); jhdeng1998@126.com (J.D.)

**Keywords:** maize, aflatoxin B_1_, near-infrared spectroscopy, Markov transition field, convolutional neural network

## Abstract

This work provides a novel approach to monitor the aflatoxin B_1_ (AFB_1_) content in maize by near-infrared (NIR) spectra-based deep learning models that integrates Markov transition field (MTF) image coding and a convolutional neural network (CNN) strategy. According to the data structure characteristics of near-infrared spectra, new structures of one-dimensional CNN (1D-CNN) and two-dimensional MTF-CNN (2D-MTF-CNN) were designed to construct a deep learning model for the monitoring of AFB_1_ in maize. The results obtained showed that compared with the 1D-CNN model, the performance of the 2D-MTF-CNN model had been significantly improved, and its root mean square error of prediction, coefficient of predictive determination, and relative percent deviation were 1.3591 μg·kg^−1^, 0.9955, and 14.9386, respectively. The results indicate that the MTF is an effective data encoding technique for converting one-dimensional spectra into two-dimensional images. It more intuitively reflects the intrinsic characteristics of the NIR spectra from a new perspective and provides richer spectral information for the construction of deep learning models, which can ensure the detection accuracy and generalization performance of deep learning quantitative detection models. This study provides a new analytical perspective for the chemometrics analysis of the NIR spectroscopy.

## 1. Introduction

During growth, fungi generate secondary metabolites such as mycotoxins, which are widely found in food crops such as maize, barley, oats, wheat, rice, and sorghum [1]. The fungal toxins with greater impact on human life and health include aflatoxin B_1_ (AFB_1_), zearalenone (ZEN), deoxynivalenol (DON), T-2toxin (T-2), fumonisin (FB), and ochratoxin A (OTA), etc. [2]. Among them, the AFB_1_ is often produced by fungi when food products are stored, and has strong carcinogenicity [3]. According to the FAO, 25% of the world’s agricultural products are contaminated with mycotoxins every year, causing hundreds of billions of dollars in economic losses [4]. China is also one of the countries with serious AFB_1_ pollution, especially in maize, wheat, and their cereal products. In order to protect the health of consumers, China has limited the content of the AFB_1_ in grain and their processed products at 5 μg·kg^−1^. Therefore, it is of great importunacy to achieve the trace and efficient monitoring of the AFB1 in grains and their products.

At present, a lot of research reports on the detection of the AFB_1_ in agricultural products and food have been reported, mainly including high-performance liquid chromatography–mass spectrometry [5], high-performance liquid chromatography [6], gas chromatography [7], etc. Although chromatography and its combined techniques can detect high sensitivity and repeatability and can achieve accurate analysis of mycotoxins in agricultural products and foods, instrumental analysis requires the training of professional technicians, complex sample pretreatment steps, and expensive equipment. It cannot meet the needs of rapid on-site detection. The basic principle of an immunoassay is the specific binding reaction of antigen and antibody. Currently, the most widely used immunoassays are the enzyme-linked immunosorbent assay (ELISA) [8] and the gold immunochromatography assay (GICA) [9]. However, these methods all require the use of chemical reagents and depend on the specificity of antigens and antibodies. The operation steps are cumbersome and time-consuming, which limits their application in on-site rapid detection. Therefore, it is urgent to develop a green, fast, and effective on-site analytical tool to realize the trace determination of the AFB_1_ in grains and their products with high precision.

Near-infrared (NIR) spectroscopy is an efficient and fast modern analysis technology, which combines computer technology, spectroscopy technology, chemometrics, and other disciplines, and has been widely used in many fields with its unique advantages [10,11,12]. In terms of food safety detection, Jiang et al. used the NIR spectroscopy to quantitatively detect the AFB_1_ in mildewed wheat [13]; Gaspardo et al. used Fourier-transform near-infrared spectroscopy to rapidly detect FB_1_ and FB_2_ in maize flour [14]; and De Girolamo et al. used infrared spectroscopy to rapidly screen OTA in wheat [15]. Although the technique has been successfully applied in the monitoring of mycotoxins in grains and their products, there are still shortcomings: (1) The acquisition of high-quality spectral data. The NIR spectroscopy has the characteristics that the sample data contain noise and the dimension of the spectral data itself is too high, which brings inconvenience to the establishment of subsequent quantitative models. (2) The selection of a suitable quantitative calibration model. Therefore, the pretreatment of spectral data, the selection of effective wavelengths, and a reasonable calibration model are the keys to the successful application of the NIR spectroscopy in trace and even trace target attribute detection.

Generally speaking, the purpose of spectral pretreatment is to eliminate the interference information and noise data information in the data. At present, the commonly used data pretreatment algorithms mainly include a smoothing algorithm, a derivative algorithm, and multiple scatting correction, etc. [16]. The selection of the characteristic wavelengths can simplify the complexity of the calibration model and reduce the computational cost. The selection algorithms for spectral data features mainly include competitive adaptive reweighted sampling, random frog leaping, and a projection algorithm, etc. [17]. On the whole, the above methods can achieve the requirements of eliminating redundant information and improving the accuracy of the calibration model. However, at present, there is no uniform rule to choose the pretreatment method of the spectra, and it can only rely on repeated attempts. In addition, the selection algorithm for spectral data features often depends on the algorithm design, which is also a drawback of the existing feature wavelength selection algorithm. Therefore, if a reliable mathematical model with high noise tolerance and autonomous processing of high-dimensional data can be found, it will largely make up for the shortcomings of the current development of spectral chemometrics.

Deep learning is a discipline that studies the inherent laws and representation levels of sample data, and has been widely used in many fields, especially in image processing [18], natural language processing [19], and data mining [20]. In fact, deep learning has been used for spectral noise suppression, feature extraction, and model calibration [21]. Xu et al. used deep learning algorithms for the accurate spectral classification of six different items [22]; Cheng et al. used the NIR spectroscopy combined with deep learning algorithms to rapidly detect cumin and fennel [23]; Yang et al. used the combination of a convolutional neural network and a recursive neural network to predict soil properties of the NIR spectra [24]. Zhu et al. proposed a new method for aflatoxin B_1_ (AFB_1_) detection inspired by quantitative remote sensing [25]. Yang et al. used hyperspectral imaging (HSI) combined with the deep stacked sparse auto-encoders (SSAE) algorithm to recognize the early mildewed degree of kernels [26]. Han et al. realized pixel-level aflatoxin detection based on deep learning and hyperspectral imaging [27]. Although deep learning algorithms have been incorporated into the field of spectral analysis, most of the existing research uses deep learning for qualitative analysis [28], such as variety identification, origin identification, and adulteration identification, and relatively few in quantitative analysis. However, there are few reports in the literature on the spectral detection of mycotoxins in grains and their products. In addition, the current spectral analysis research based on deep learning mainly focuses on the one-dimensional data level [29,30], and the deep learning processing based on spectral image-level has not been reported yet.

Thus, the main work arrangements of this study are as follows: (1) Use the team’s self-made portable near-infrared spectroscopy system to collect spectral data on maize with different degrees of mildew; (2) Simulate data augmentation methods, adding different degrees of noise to the original spectral data to expand the sample library; (3) Use the Markov transition field method to transform the spectral data into two-dimensional images to build a deep learning model to achieve efficient monitoring of the AFB_1_ content in maize, and compare the capabilities of this model with that of the deep learning model based on one-dimensional spectral data.

## 2. Materials and Methods

### 2.1. Sample Preparation

The maize utilized in this work were 10 kg of fresh bulk maize pellets purchased from a supermarket in Zhenjiang, Jiangsu Province. In order to simulate the growth environment of mold during the mildew process of maize, some of the purchased maize samples were placed in a constant temperature and humidity incubator (HWS-250B, Hongnuo Instrument Co., Ltd., Tianjin, China). The temperature and humidity of the constant temperature and humidity incubator were set at 30 ± 3 °C and 80–90%, respectively, to accelerate the mildew process of maize. In addition, to ensure that this environment remained consistent throughout the experiment, water mist was sprayed into it periodically. Finally, during the experiment phase, 120 samples were obtained by six random sampling sessions, with an interval of 3–5 days between each sampling session, each sampling being 20 samples, each weighing 20 g.

### 2.2. Detection of Aflatoxin B_1_ Content

The method studied for the determination of AFB_1_ content in maize flour samples was a competitive colloidal gold technique, which has made great achievements in the rapid detection of toxins. The specific experimental steps were as follows: First, 70% methanol was poured into maize samples to extract the AFB_1_ from the sample. Then, 100 μL of the supernatant from the extract after shaking and centrifugation was extracted, and 400 μL of dilution was added and mixed. Finally, 90 μL of the mixture was taken to drop it to the test card, and the result was recorded.

### 2.3. Spectral Acquisition

For the acquisition of NIR spectra of all samples, the work was conducted using a handheld NIR spectrometer constructed by our group. The wavelength range of the instrument is 901.78-1661.24 nm with the optical resolution of 10 nm.

**Figure 1:**
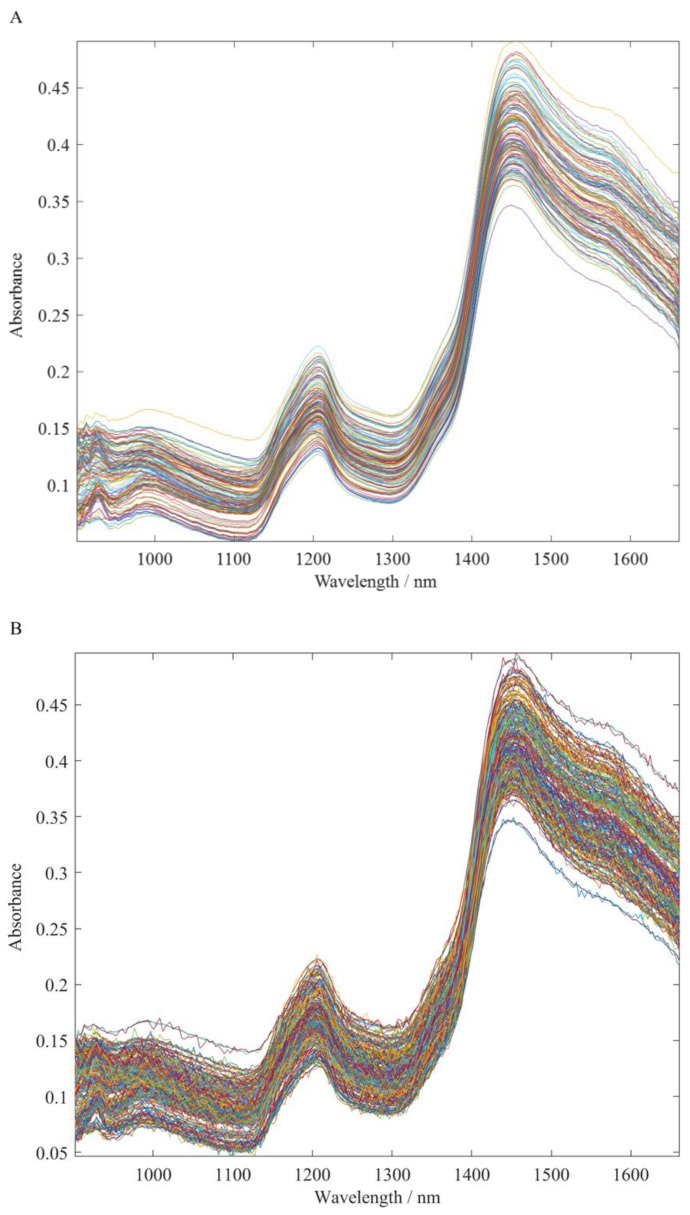
NIR spectra (**A**) and the NIR spectra by data augmentation (**B**) of all samples.

Before spectral collection, the sample was ground into powder using a multifunctional grinder (BJ-150, Baijie Electrical Co., Ltd., Deqing, China). In addition, for each sample, the mean of three sampling spectra was taken as the original spectrum of the sample. Figure 1A presents the raw spectra of all samples.

### 2.4. Data Analyses Methods

#### 2.4.1. Spectral Augmentation

Deep learning is essentially a process of autonomous learning of the features and rules in data sets. The parameters that need to be learned will increase with the number of neural network layers, which will make it more prone to overfitting. It is of concern that when the data set involved in training is small, too many parameters will fit all the characteristics of the data set instead of the common characteristics among the data, which is a problem that should be avoided in chemical analysis. Therefore, taking appropriate measures to expand the data set before network training is one of the ways to avoid overfitting. In this case, data augmentation techniques are a good choice [31].

In this study, we augment the original spectral data by adding noise with different degrees of signal-to-noise ratios to the original spectral data. In fact, this method is feasible. This is mainly because it not only simulates experimental samples to obtain noisy data under different factors, but also further verifies the feasibility of the network we built for spectral analysis. Here, we used the built-in function “awgn” in MATLAB to augment the original NIR spectra obtained by adding white noise to the data. Figure 1B shows the spectral image after adding noise and all spectral data after data augmentation (120 × 5). During data enhancement in this study, the signal-to-noise ratios were set to 80 dB, 70 dB, 60 dB, and 50 dB, respectively.

#### 2.4.2. Markov Transition Field

Deep learning has made great progress and development in the area of computer vision. In order to make full use of the advantages of deep learning in the area of image processing, it is necessary to study the method of converting a one-dimensional spectral sequence into a two-dimensional image, and then use a convolutional neural network for subsequent quantitative analysis. Taking advantage of the similarity between spectral data and time series, that is, the NIR spectral sequence based on wavelength points, the correlation between wavelengths cannot be ignored. Based on this, this study uses the Markov transition field (MTF) image coding algorithm to map one-dimensional spectral data into two-dimensional image data [32]. The specific implementation steps are as follows: The first step is to obtain the original near-infrared spectral data of the sample and perform normalization processing. Let the spectral sequence of each sample be $X=\left\{x_{1},x_{2},x_{3},\ldots ,x_{n}\right\}$, n is the total number of wavelength points. The second step is to define quantiles for the spectral series. Given a spectral sequence *X*, define its *Q* (quantile), and assign $x_{i}$ in each spectral sequence to the corresponding quantile $q_{j}$ ($j\in \left[1,Q\right]$). The third step is to generate a *Q × Q* Markov transition matrix based on the *Q* quantile. By calculating the transition probability between the first-order Markov chains, the following matrix is finally obtained.

(1) $$M=\left[\begin{array}{ccc} w_{ij|x_{1}\in q_{i},x_{1}\in q_{j}} & \cdots & w_{ij|x_{1}\in q_{i},x_{n}\in q_{j}}\\ w_{ij|x_{2}\in q_{i},x_{1}\in q_{j}} & \cdots & w_{ij|x_{2}\in q_{i},x_{n}\in q_{j}}\\ \ldots & \ddots & \ldots \\ w_{ij|x_{n}\in q_{i},x_{1}\in q_{j}} & \cdots & w_{ij|x_{n}\in q_{i},x_{n}\in q_{j}} \end{array}\right]$$

where, $q_{i}$ and $q_{j}$ are the quantiles corresponding to $x_{i}$ and $x_{j}$, respectively.

#### 2.4.3. Convolution Neural Network

A convolutional neural network, as one of the important algorithms in the field of deep learning, has the advantages of sharing the receptive domain and weights, reducing the number of neural network parameters to be trained, and simplifying the complexity of the model compared with the traditional artificial neural network [33]. It has unique advantages in image processing, target detection, target tracking, and so on. In recent years, CNN learning algorithms have been successfully applied in many fields, especially in the field of analytical chemistry [34].

**Figure 2:**
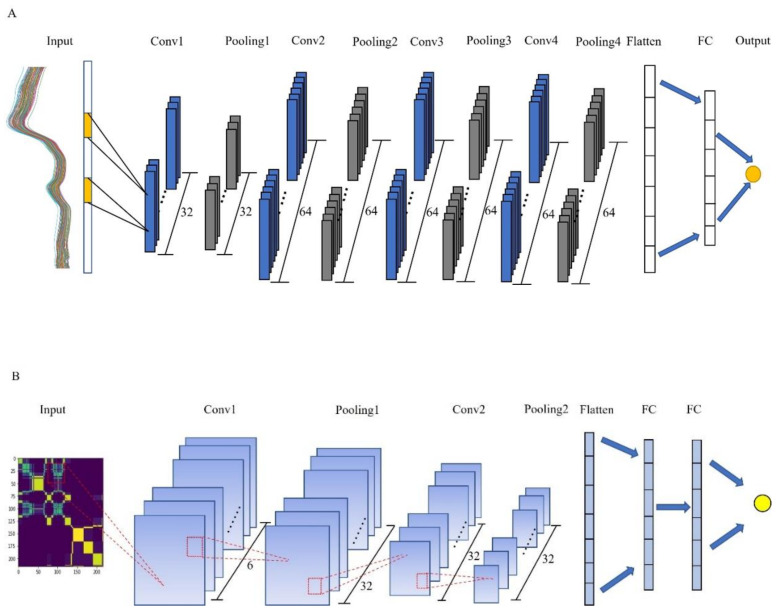
The designed structure of convolutional neural network models. (**A**) 1D-CNN; (**B**) 2D-MTF-CNN.

A complete CNN usually consists of four main components: a convolutional layer, an activation layer, a pooling layer, and a fully connected layer [35]. The convolutional layer is composed of a series of convolution kernels, and the convolutional layer undertakes the heavy task of extracting the features of the input image in the whole network. Starting from the basic features such as edges and shapes of the initial layer, as the depth of the network deepens, the later convolution layers will get more complex and specific features. The activation layer is generally located after the convolution layer and the full connection layer, and the results are mapped nonlinearly. A pooling layer is used to reduce the dimensionality of the feature vector and a fully connected layer is used to achieve the final prediction or classification. Figure 2 shows the one-dimensional and two-dimensional convolution neural network structures designed to achieve efficient monitoring of the AFB_1_ in maize.

### 2.5. Figures of Merit

In this study, the root mean square error of cross-validation (RMSECV) and the coefficient of correction determination ($R_{C}^{2}$) were used to evaluate the detection accuracy of different PLS models, and the root mean square error of prediction (RMSEP) and coefficient of predictive determination ($R_{P}^{2}$) to evaluate the generalization performance of different PLS models.

## 3. Results

### 3.1. Division of Calibration Set and Prediction Set

In this study, a total of 600 sample data were obtained through data augmentation. To ensure a reasonable data allocation, both datasets must contain measurements of the AFB_1_ content for all gradients as well as sample data with varying degrees of noise. Therefore, the sample set is divided based on the following. First, the sample data without added noise are sorted according to the AFB_1_ measurements from smallest to largest. Here, we set the expected output of the newly generated spectra to be the same as the AFB_1_ concentrations of the corresponding original spectra. Then, one of every four samples was randomly selected to be put into the prediction set and the other three samples into the calibration set. Thus, the calibration set had 450 sample data, of which 75% (405 sample data) were used to train the network, and the remaining 45 sample data were used to verify the trained network. In the prediction set, there were 150 samples (25%), which were used to test the generalization performance and stability of the training network. The statistical result of the AFB_1_ content of all samples in the two sample sets is shown in Table 1.

### 3.2. The Training Results of CNN Models

**Figure 3:**
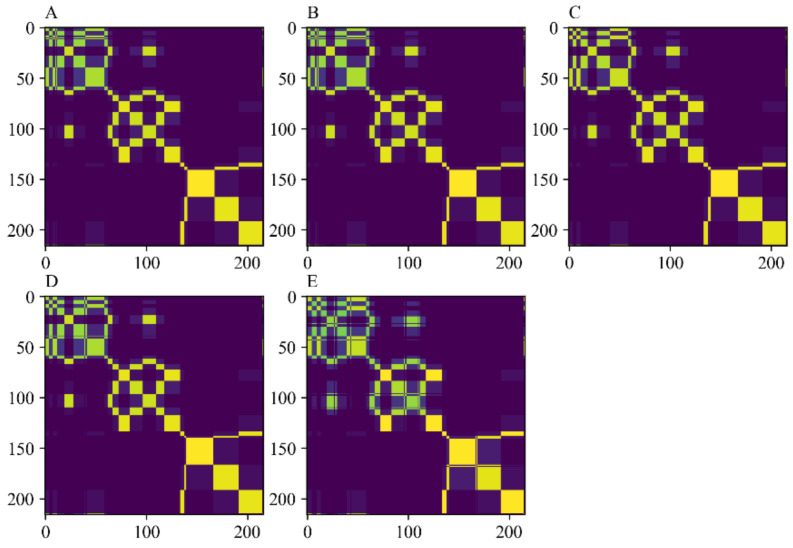
The images of Markov transition field with different noise levels. (**A**) No noise added; (**B**) Add a noise level of 80 dB; (**C**) Add a noise level of 70 dB; (**D**) Add a noise level of 60 dB; (**E**) Add a noise level of 50 dB.

In this work, firstly, an attempt was made to establish a detection model for the prediction of AFB_1_ content in maize samples applying a one-dimensional convolutional neural network (1D-CNN). In order to reflect the advantages of the deep learning algorithm in spectral analysis, a unique normalized pretreatment was carried out on the data after data augmentation. Secondly, in order to make use of the advantages of convolutional neural networks in the field of image processing, the MTF was used to map one-dimensional spectral data into two-dimensional images for processing. The Markov transform domain image encoding process neither loses any features of the original one-dimensional spectrum, but also realizes the bidirectional mapping from one-dimensional spectral signals to two-dimensional images. Using this method, the spectral data of the calibration set and the prediction set were encoded in two-dimensional images, and finally compressed in the specified format for the training of the network model. Figure 3 shows a Markov transform domain image containing spectra with five different levels of noise.

The purpose of establishing the CNN model is to enable the spectral feature points (215 feature points in total) to fully reflect the AFB_1_ content. The designs of two different dimensional CNN models differ in different types of layers. Table 2 shows the specific structure and parameter settings of each layer. Both models are determined through repeated debugging under the condition of comprehensive consideration of overfitting and underfitting. Part of the training parameters was set as follows: the root mean square error (RMSE) was taken as the loss function, the determination coefficient was taken as the performance evaluation index of the model, and Adam with an initial learning rate of 0.001 was taken as the optimization algorithm. In addition, for the one-dimensional convolutional data network, the number of training batch samples was set to batch_size = 150, and the training round epoch = 3000. The number of two-dimensional convolutional neural training batches was set to batch_size = 50, and the training round epoch = 300.

**Figure 4:**
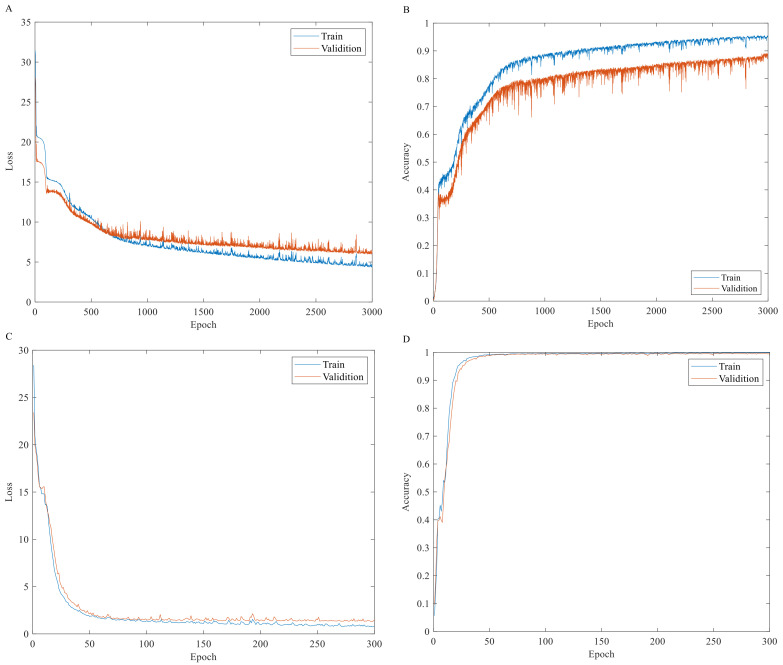
The training results of the convolutional neural network. (**A**) Loss of 1D-CNN; (**B**) Accuracy of 1D-CNN; (**C**) Loss of 2D-MTF-CNN; (**D**) Accuracy of 2D-MTF-CNN.

Figure 4 shows the training process of two convolutional neural networks. It is not difficult to find from Figure 4 that the loss function of the two models decreases with the increase in training times, while the accuracy increases with the increase in training times. This shows that both networks are actively learning the features of spectral data during network training. Further analysis of Figure 4A,B shows that in the training process of the 1D-CNN, when the number of training times is 1500, the variation of loss function and accuracy tends to be flat, and the accuracy of the calibration set and the validation set shows a big difference, indicating that the model is slightly underfitting due to overlearning in the training process. The analysis of Figure 4C,D shows that the most stable point during the training of the 2D-MTF-CNN appears around the 100th time, which is consistent with the optimal loss value. The analysis of Figure 4D shows that with the increase in iteration times, the training accuracy converges rapidly and approaches one. Regardless of the loss function or the accuracy, in the subsequent iterations, the curves of two sample sets are basically the same, which indicates that the 2D-MTF-CNN training model is more stable than the 1D-CNN model and has a better detection performance.

## 4. Discussion

The statistics of the prediction results of the 1D-CNN model and the 2D-MTF-CNN model for 150 independent samples in the prediction set are shown in Table 3. From Table 3, the $R^{2}$ of the 1D-CNN and the 2D-MTF-CNN model are both above 0.90, and both have a good prediction accuracy and generalization performance. In addition, the overall performance of the 2D-MTF-CNN model are significantly improved compared to the 1D-CNN model, the $R_{P}^{2}$ value is increased from 0.9227 to 0.9955, the RPD value is increased from 3.8101 to 14.9386, and the RMSEP value is reduced from 5.5360 to 1.3591 μg·kg^−1^. The above results verify that when using deep learning to process NIR spectral data, it is not necessary to perform preprocessing and feature optimization on the original spectra, but directly perform multivariate calibration on the spectra to build a quantitative analysis model and obtain better results. In fact, in this study, we are not surprised by the results achieved. First, for the whole spectral data set, there are similarities and differences between the spectra of different toxin concentrations, which are reflected in the positions and intensities of the characteristic peaks of the spectra. For some of the samples, which have similar toxin concentrations, the characteristic peaks and intensities of the spectra are difficult to distinguish. This can lead to errors in the prediction of unknown samples, resulting in a lower prediction accuracy. After the spectrum is encoded by the MTF image, the characteristic information of the original spectrum is retained and its characteristic information is enhanced, which is easier for the network to learn. This phenomenon can be seen from the structure of the MTF-CNN network; that is, fewer convolutional layers are used for feature extraction of images to achieve better results.

**Figure 5:**
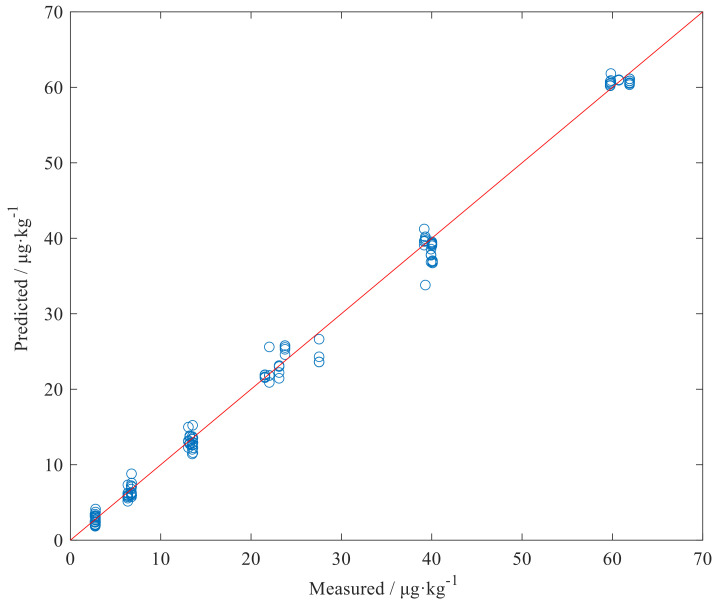
Comparison of measured and the 2D-MTF-CNN model predicted values.

Therefore, we can draw the following conclusion that the 2D-MTF-CNN model proposed in the research can be viewed as a high-precision model to monitor the AFB_1_ content in maize. The scatter diagram between the predictive results of the 2D-MTF-CNN model in the prediction set and the measured reference value is shown in Figure 5.

## 5. Conclusions

This investigation verifies the feasibility of using the NIR spectra to build a deep learning model to achieve efficient monitoring of the AFB_1_ content in maize. According to the structural characteristics of the NIR spectral data, the models were created by designing deep learning networks with 1D-CNN and two-dimensional 2D-MTF-CNN structures, respectively. The results revealed that the coefficients of determination for the calibration and the prediction of the AFB_1_ content in maize were both above 0.90 by 1D-CNN and 2D-MTF-CNN models. Furthermore, the detection performance of the 2D-MTF-CNN model is significantly improved compared to the 1D-CNN model. Therefore, we can infer that MTF is an effective near-infrared spectral data encoding technology, which can successfully convert one-dimensional NIR spectral data into two-dimensional image data, providing a reliable data basis for the construction of high-precision deep learning models. This study provides a technical reference for the mature application of deep learning theory in chemometric analysis of the NIR spectroscopy.

## Figures and Tables

**Table 1:** Statistical results of the AFB_1_ value of peanut oil samples in calibration set and the prediction set.

Sample Sets	Sample Number	Maximum/μg·kg^−1^	Minimum/μg·kg^−1^	Mean/μg·kg^−1^	Standard Deviation/μg·kg^−1^
Calibration set	450	63.0195	2.6214	24.4588	20.4806
Prediction set	150	61.9111	2.7252	24.4746	20.3720

**Table 2:** The structures and parameters of the 1D-CNN and 2D-MTF-CNN models.

Models	Layers	Size	Number	Activation	Output Shape	Parameters
1D-CNN	Input	(215,1)	-	-	-	-
	Conv1	3×1	32	Relu	(213,32)	128
	Max pooling	3×1	-	-	(71,32)	0
	Conv2	3×1	64	Relu	(69,64)	6208
	Max pooling	3×1	-	-	(23,64)	0
	Conv3	3×1	64	Relu	(21,64)	12,352
	Max pooling	3×1	-	-	(7,64)	0
	Conv4	3×1	64	Relu	(5,64)	12,352
	Max pooling	2×1	-	-	(2,64)	0
	Flatten	-	-	-	128	0
	Dense	1	-	Linear	1	129
2D-MTF-CNN						
	Input	(215,215,1)				
	Conv1	11×11	6	Relu	(206,206,6)	732
	Max pooling	2×2	-	-	(103,103,6)	0
	Conv2	11×11	32	Relu	(93,93,32)	23,264
	Max pooling	3×3	-	-	(31,31,32)	0
	Flatten	-	-	-	30,752	0
	Dense1	10	-	Relu	10	307,530
	Dense2	10	-	Relu	10	110
	Dense3	1	-	Relu	1	11

**Table 3:** Raman characteristic peak attribution.

**Models**	**Input Shape**	RMSEC/μg·kg^−1^	$R_{C}^{2}$	RMSEP/μg·kg^−1^	$R_{P}^{2}$	RPD
1D-CNN	(215,1)	3.7397	0.9637	5.5360	0.9227	3.8101
2D-MTF-CNN	(215,215,1)	0.6799	0.9989	1.3591	0.9955	14.9386

## Data Availability

The data is currently classified and will be available in 2024 with permission from the project.
